# Locating the Site of Neuropathic Pain *In Vivo* Using MMP-12-Targeted Magnetic Nanoparticles

**DOI:** 10.1155/2019/9394715

**Published:** 2019-03-06

**Authors:** Syeda Fabeha Husain, Raymond W. M. Lam, Tao Hu, Michael W. F. Ng, Z. Q. G. Liau, Keiji Nagata, Sanjay Khanna, Yulin Lam, Kishore Bhakoo, Roger C. M. Ho, Hee-Kit Wong

**Affiliations:** ^1^Department of Psychological Medicine, Yong Loo Lin School of Medicine, National University of Singapore, Singapore 119228; ^2^Biomedical Institute for Global Health Research and Technology, National University of Singapore, Singapore 117599; ^3^Department of Orthopaedic Surgery, Yong Loo Lin School of Medicine, National University of Singapore, Singapore 119228; ^4^Department of Spine Surgery, Shanghai East Hospital, Tongji University School of Medicine, Shanghai, 200120, China; ^5^Singapore Bioimaging Consortium, Singapore 138667; ^6^Department of Orthopaedic Surgery, National University Hospital, National University Healthy System, Singapore 119228; ^7^Department of Physiology, Yong Loo Lin School of Medicine, National University of Singapore, Singapore 117593; ^8^Department of Chemistry, National University of Singapore, Singapore 117543; ^9^Centre of Excellence in Behavioural Medicine, Nguyen Tat Thanh University, Ho Chi Minh City 70000, Vietnam; ^10^Faculty of Education, Huaibei Normal University, Huaibei, Anhui 235000, China

## Abstract

Neuropathic pain remains underrecognised and ineffectively treated in chronic pain sufferers. Consequently, their quality of life is considerably reduced, and substantial healthcare costs are incurred. The anatomical location of pain must be identified for definitive diagnosis, but current neuropsychological tools cannot do so. Matrix metalloproteinases (MMP) are thought to maintain peripheral neuroinflammation, and MMP-12 is elevated particularly in such pathological conditions. Magnetic resonance imaging (MRI) of the peripheral nervous system has made headway, owing to its high-contrast resolution and multiplanar features. We sought to improve MRI specificity of neural lesions, by constructing an MMP-12-targeted magnetic iron oxide nanoparticle (IONP). Its *in vivo* efficiency was evaluated in a rodent model of neuropathic pain, where the left lumbar 5 (L5) spinal nerve was tightly ligated. Spinal nerve ligation (SNL) successfully induced mechanical allodynia, and thermal hyperalgesia, in the left hind paw throughout the study duration. These neuropathy characteristics were absent in animals that underwent sham surgery. MMP-12 upregulation with concomitant macrophage infiltration, demyelination, and elastin fibre loss was observed at the site of ligation. This was not observed in spinal nerves contralateral and ipsilateral to the ligated spinal nerve or uninjured left L5 spinal nerves. The synthesised MMP-12-targeted magnetic IONP was stable and nontoxic *in vitro*. It was administered onto the left L5 spinal nerve by intrathecal injection, and decreased magnetic resonance (MR) signal was observed at the site of ligation. Histology analysis confirmed the presence of iron in ligated spinal nerves, whereas iron was not detected in uninjured left L5 spinal nerves. Therefore, MMP-12 is a potential biomarker of neuropathic pain. Its detection *in vivo*, using IONP-enhanced MRI, may be further developed as a tool for neuropathic pain diagnosis and management.

## 1. Introduction

Back pain is the highest reported pain condition and progresses to chronic lower back pain in two-thirds of patients. These patients present nociceptive and/or neuropathic components, but the later component is usually underrecognised [1]. Neuropathic pain is defined as pain arising as a direct consequence of a lesion or disease, affecting the somatosensory system. Diagnosis requires identifying the anatomical location and pathology. However, the most convenient methods of documenting patient history, and physical examination, rely on patient collaboration [2]. Since patients' experience of pain is influenced by cognitive, emotional, and educational factors, more objective measurements of neuropathic pain are called for. Current screening tools to assess nerve damage are neuropsychological in nature, which include questionnaires and various electrodiagnostic tests. However, they cannot provide conclusive evidence for neuropathic pain or locate the exact pain-generating site [3].

MRI techniques are increasingly applied to observe neural injuries and diseases noninvasively. In T2-weighted images (T2-WI), peripheral nerves are normally indistinguishable from surrounding tissues and may appear slightly hyperintense, or isointense, relative to muscle tissue. However, injured nerves appear bright on T2-WI, as the T2 relaxation time is prolonged [4]. Although MR signal changes in injured peripheral nerves are significant, it lacks specificity. Regenerating nerves cannot be distinguished from chronically degenerating ones [5], and the same signal changes may be observed for normal nerves [6]. MRI contrast agents, such as gadolinium [7, 8], manganese [9], perfluorocarbon [10], and IONP [11–13], were developed to address this gap. However, contrast agents used thus far in patients and animal models are nonspecific. Hence, in the present study, we sought to design an IONP-based MR contrast agent that would be selectively cleaved and taken up locally by injured nerve roots.

IONP markedly decreases T2 signals by enhancing the spin-spin relaxation times of surrounding water protons [14]. IONP has been used to observe macrophage migration into experimentally injured sciatic nerves *in vivo* [11, 13]. Bendszus and Stoll [13] reported IONP-induced signal loss up to 8 days after sciatic nerve injury. However, the signal increases by day 11 because iron-laden macrophage recruitment is transient, and infiltrated macrophages remain stationary within the injured nerve [13]. IONP can detect enzymatic activity, when conjugated to peptide sequences. When the peptide sequence is cleaved by the extracellular target enzyme, the released IONP would be taken up by surrounding tissues [15–18]. Unlike nontargeted IONPs, targeted IONPs may prevent damage to other tissues, as they would only be absorbed by those expressing the targeted enzyme [19]. Hence, macrophage factors, including MMPs, may instead present as potential targets for *in vivo* IONP-based MRI.

MMPs are a family of calcium dependent zinc endopeptidase, which are responsible for extracellular matrix (ECM) degradation. Their activity is tightly controlled under physiological conditions. Upon nerve damage, MMPs degrade blood-nerve barrier and myelin. In addition, they exacerbate leukocyte infiltration and cytokine release. Hence, MMPs are thought to maintain inflammatory pathologies such as peripheral neuropathy [20]. Certain MMPs, such as MMP-12, maintain low expression in undamaged sciatic nerves. However, upon nerve injury, there is increased expression, which remains elevated for up to 20 days after injury [21, 22]. We hypothesise that elevated MMP-12 activity at the site of nerve injury can be detected *in vivo*. An MMP-12-targeted magnetic IONP may be able to locate the pain-generating site by MRI. Thus, we sought to evaluate an MMP-12-targeted IOPN in a rat SNL model. This particular model better recapitulates partial nerve injury seen in most neuropathic pain patients [23]. The first aim was to determine the effect of SNL on mechanical withdrawal threshold (MWT), thermal withdrawal latency (TWL), macrophage infiltration, MMP-12 expression, myelination, and elastin fibre content. The second aim was to evaluate the cytotoxicity, cellular uptake, and specificity, of the synthesised MMP-12-targeted magnetic IONP, *in vitro*. The last aim was to examine *in vivo* MRI of animals receiving the MMP-12-targeted magnetic IONP and to verify IONP uptake by histology analysis of spinal nerves.

## 2. Materials and Methods

### 2.1. Animals

Procedures involving animals were approved by the Institutional Animal Care and Use Committee, of the National University of Singapore (R13-4649, R14-0009), and carried out in accordance with the National Advisory Committee for Laboratory Animal Research guidelines. 7-week-old male Sprague–Dawley rats (InVivos, Singapore), initially weighing 200 to 250 g, were housed under controlled temperature (20–26°C) and humidity (30–70%), with a 12-hour light-dark cycle (lights on 7:00 AM to 7:00 PM). Food and water were provided *ad libitum*. Animals underwent either SNL or sham surgery. They were assessed for mechanical allodynia and thermal hyperalgesia, by von Frey and Hargreaves' tests, respectively. These tests were done 3 days preoperatively, as well as 7 and 13 days postoperatively. Spinal nerves harvested from rats euthanized 1 or 2 weeks after SNL or sham surgery were studied by immunohistochemistry and histology staining or MMP-12 activity assay. Rats that underwent intrathecal injection of MMP-12-targeted IONP, 2 weeks after SNL or sham surgery, were euthanized after MRI.

### 2.2. Surgical Procedures

All surgical procedures were performed after subcutaneous administration of enrofloxacin (10 mg/kg). Rats were deeply anesthetised with isoflurane (5% induction and 2% maintenance in air) and placed in a prone position. The dorsal thoracic area was shaved of fur, and the skin was scrubbed thrice, alternating between iodine and 70% ethanol, before animals were draped.

SNL surgery began by making a dorsal midline incision. The left paraspinal muscles were separated from the spinous processes by sharp and blunt dissection, to expose the left L6 transverse process. The left L6 transverse process was carefully removed using a bone trimmer, to expose the left L5 spinal nerve. The nerve was tightly ligated with a 5-0 chromic gut suture, 3–5 mm distal to the dorsal root ganglion (DRG). Muscle and skin were closed with 3-0 absorbable suture. Lignocaine (10 mg/ml, Pfizer) was injected under the sutured skin, and Banocin powder was applied on the sutured skin. In sham surgery, the left L5 spinal nerves are exposed but not ligated [24]. To inject the MMP-12-targeted probe, the L5 vertebrae were exposed in a similar manner. The laminar bone was minimally removed using a rongeur, to allow intrathecal injection of 10 *μ*l of the MMP-12-targeted IONP onto the left L5 DRG with a GC syringe [25].

After any surgery, animals were housed individually with clean bedding and treated with subcutaneous enrofloxacin twice daily for 3 days. Those injected with MMP-12-targeted probe were also given buprenorphine (0.06 mg/kg) twice daily for 3 days. Animals were monitored for signs of distress after any surgery. There were no instances of neurological deficit or paralysis due to SNL throughout the course of this study.

### 2.3. Behavioural Assessments

Mechanical allodynia was evaluated using von Frey filaments, in an automated Dynamic Plantar Aesthesiometer system (UgoBasile, Italy). The system is placed under an elevated wire mesh, and rats are placed singly in transparent boxes upon the mesh. For every trial, the filament is carefully positioned under the plantar surface of the hind paw and rises to press against the paw. The applied force ranged from 5 g to 40 g, for a maximum of 6 seconds or until the paw is withdrawn. MWT is reached at a force, when hind paws are withdrawn in under 6 seconds, for at least 5 out of 6 trials. Thermal hyperalgesia was evaluated by Hargreaves' method using the thermal plantar test apparatus (UgoBasile, Italy). This system is placed below an elevated plastic platform, and rats were placed singly in transparent boxes upon this platform. For every trial, the infrared source (40 mW) is positioned beneath the midplantar surface of the hind paw, for a maximum of 20 seconds or until the paw was withdrawn [26]. The average withdrawal time of five trials, where the paw is withdrawn in under 20 seconds, was calculated as the TWL. The MWT and TWL difference was calculated for each rat, by subtracting the MWT or TWL of the left hind paw from that of the right hind paw [24].

### 2.4. Histology and Immunohistochemistry

Specimens were obtained from animals euthanized by terminal cardiac perfusion. These rats were deeply anesthetised with 1 ml urethane and perfused with 400 ml 1% sodium nitrite, followed by 400 ml 4% paraformaldehyde (PFA). The proximal and distal lengths, between the DRG, and each end of the excised spinal nerves, were 5–7 mm. The harvested left L4, L5, and L6, and right L5 spinal nerves were placed in 4% PFA and processed for paraffin embedding. Before any staining, 5 *μ*m thick sections were deparaffinised by xylene and hydrated using graded ethanol and distilled water.

Immunohistochemistry began with antigen retrieval using pepsin for 15 min at 37°C. Endogenous peroxidase activity was blocked with 1% hydrogen peroxide for 10 min, followed by avidin and biotin block (SP-2001, Vector Laboratories, CA, USA) for 15 min each. Sections were incubated with 1.5% normal goat serum (AK-5001, Vector Laboratories, CA, USA), before a 2 h incubation with either a mouse monoclonal antibody to CD68 (MAB1435, Merck Millipore, MA) or a rabbit polyclonal antibody to MMP-12 (bs-1854R, Bioss, MA, USA), at a dilution of 1 : 200. biotinylated goat anti-mouse (Ba-2900, Vector Laboratories, CA, USA) or anti-rabbit (AK-5001, Vector Laboratories, CA, USA) IgG antibodies diluted by 1 : 200 in 1.5% normal goat serum were applied for 30 min. Thereafter, an avidin-biotin complex reagent (AK-5001, Vector Laboratories, CA, USA) was applied for 30 min, followed by visualisation using ImmPACT Vector Red Alkaline Phosphatase (SK-5105, Vector Laboratory, CA, USA) for 20 min. Slides were counterstained in hematoxylin (3801562, Leica Biosystems Inc, IL, USA) for 1 min.

In Verhoeff-van Gieson (ab150667, Abcam, United Kingdom) staining, slides were immersed in working elastic stain solution for 8 min, dipped in differentiating solution 40 times, placed in sodium thiosulfate solution for 1 min, and rinsed in tap water after every reagent. They were then stained with van Gieson's solution for 30 seconds and rinsed in 95% ethanol for 30 seconds.

Luxol Fast Blue staining was carried out after hydrating sections to 95% ethanol. Slides were immersed in 0.1% Luxol Fast Blue (S3382-25G, Sigma-Aldrich, MO, USA) solution for 16 h in a 57°C water bath. After brief washing in 95% ethanol and distilled water, sections were differentiated in 0.05% lithium carbonate (152,537, MP Biomedicals, CA, USA) solution for 1 min and washed with distilled water.

Prussian blue staining was done by immersing slides in a working solution of equal parts of 20% aqueous hydrochloric acid and 10% aqueous potassium ferrocyanide (P-3289, Sigma-Aldrich, CA, USA) solution for 20 min, followed by 0.1% nuclear fast red (60,700, Sigma-Aldrich, CA, USA) solution for 5 min. Slides were thoroughly washed in distilled water after every solution or stain.

All stained slides were dehydrated in graded ethanol and xylene. After mounting slides with DPX (06522, Sigma-Aldrich, CA, USA), bright field images were acquired (Olympus CX41, Japan). For each specimen, 2 sections at least 40 *μ*m apart were selected. For each section, 3 nonoverlapping images at the region of interest were taken. Using ImageJ, they were converted to greyscale images and the percentage area that was positively stained was derived. The average percentage area of 6 images per specimen was used for statistical analysis.

### 2.5. MMP-12 Activity Assay

MMP-12 concentrations in the left L4, L5, and L6, and right L5 spinal nerves, harvested from rats euthanized by carbon dioxide exposure, were determined using an MMP-12 activity assay (AS-71157, AnaSpec, CA, USA). Specimens were homogenized in assay buffer containing 0.1% Triton X-100 and centrifuged at 10000 ×g for 15 min at 4°C. The supernatant obtained was further diluted in assay buffer by 10-fold. MMP-12 concentrations were calculated relative to a human MMP-12 catalytic domain (1 *μ*g/ml, AS-55525, Anaspec, CA, USA) standard curve. 1 mM 5-FAM-Pro-Leu-OH was used as a 5-FAM fluorescence reference standard for instrument calibration. 50 *μ*l of each diluted spinal nerve specimen supernatant, human MMP-12 catalytic domain dilutions, 5-FAM-Pro-Leu-OH dilutions, and a substrate control containing assay buffer only were added to a 96-well plate. 50 *μ*l of MMP-12 substrate was added to each well before incubating at 37°C for 45 min. Thereafter, 50 *μ*l stop solution was added in all wells, and the fluorescence intensity at Ex/Em = 490/520 nm was measured (PHERAstar fluorimeter, BGM Labtech, Germany). Total protein for each sample was measured relative to an albumin standard curve, using a Micro BCA Protein Assay Kit (Micro BCA Protein Assay Kit, Thermo Scientific, MA, USA).

### 2.6. MMP-12-Targeted IONP Preparation

IONPs were synthesised and coated with dextran, according to the method described by Josephson et al. [27]. Nanoparticles were conjugated with MMP-12 probe linker, via the succinimidyl iodoacetate linker, which acts as a membrane translocator and targeting moiety (Succyl-eeeeeeeee-RPKPVE-Nva-WR-rrrrrrrr-c). Dynamic light scattering and zetapotential measurements were taken (Malvern Zetasizer Nanoseries, United Kingdom).

To determine iron concentration, the MMP-12-targeted IONP was digested in a mixture of 4 parts of 65% nitric acid to 1 part of 30% hydrogen peroxide. The digests were dried in Perfluoroalkoxy cups on a hotplate, reconstituted, and diluted in 0.4% nitric acid, to a concentration of 10–60 ng/g. Samples were analysed by graphite furnace atomic absorption spectrometry (Varian AA240Z, SpectraLab Scientific Inc., Canada), using standards prepared from Titrisol iron standard (109972, Merck, Germany) for external calibration.

### 2.7. Probe Cytotoxicity, Cell Uptake, and *In Vitro* MRI

Probe cytotoxicity was evaluated by methyl thiazolyl tetrazolium (MTT; M2128, Sigma-Aldrich, CA, USA) assay, on MMP-12-expressing U87 glioma cells [28]. A density of 1 × 10^5^ U87 glioma cells were seeded in a 96-well plate and incubated with MMP-12-targeted IONP at different concentrations (20 and 10 *μ*g/ml) for 4 h, followed by incubation with 10 *μ*l of MTT solution (5 mg/ml) for 2 h. Thereafter, the media was aspirated, dissolved in dimethyl sulfoxide, and the absorbance at 570 nm was measured. Cell viability was calculated as a percentage of absorbance in comparison with control cells.

To observe cellular uptake of the probe, U87 glioma cells were seeded into a 48-well plate, at a density of 5 × 10^3^ cells per well. Cells were incubated with 10 *μ*g MMP-12-targeted IONP and fixed with 4% PFA for 40 min. After washing cells with PBS, they were stained using the prussian blue protocol described above.

An *in vitro* MRI was done in a 7T scanner (Bruker Clinscan system, Germany) using U87 glioma cells at a density of 1 × 10^5^. They were either left untreated, incubated with MMP-12-targeted IONP for 1 h only, or incubated with the selective MMP-12 inhibitor, MMP408 (CAS 1258003-93-8, Merck Millipore, Germany) [29] for 2 h, followed by a 1 h incubation with the MMP-12-targeted IONP. After their respective treatments, cells were digested with trypsin, centrifuged, fixed with 4% PFA for 1 h at 4°C, and embedded in 1% agarose. T2-WI were acquired with the following parameters: FOV 50 mm, TR 2750 ms, TE 37 ms, and slice thickness 1 mm. The mean grey area within the region of interest (ROI) was measured using ImageJ software. MMP-12 activity of untreated U87 glioma cells, and those incubated with MMP408, was quantified using the assay described above.

### 2.8. *In Vivo* MRI

A day after intrathecal administration of MMP-12-targeted IONP, MRI was performed on rats with a 7T scanner (Siemens Magnetom ClinScan syngo, Germany). Rats were deeply anesthetised with isoflurane (5% induction and 2% maintenance in air). Temperature and respiration were monitored during T2-WI acquisition. The following parameters were used: FOV 60 mm, TR 1010 ms, TE 37 ms, slice thickness 0.8 mm, and plane resolution 0.188 × 0.188 mm.

### 2.9. Statistical Analysis

All data are expressed as mean ± standard deviation (SD) and analysed with Graphpad Prism 7 software. Behavioural, immunohistochemistry, histology, and MMP-12 activity assay data were analysed using Mann–Whitney *U* test, where *p* < 0.05 was considered statistically significant.

## 3. Results and Discussion

### 3.1. SNL Induces Neuropathic Pain

**Figure 1:**
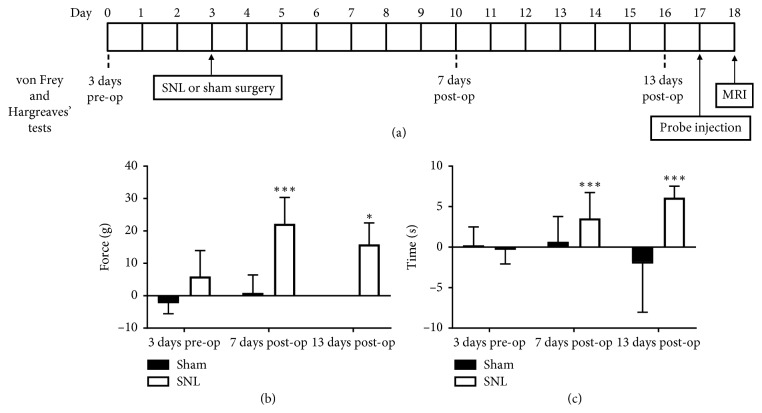
Left L5 SNL induces neuropathic pain in rats. Behavioural assessments were done (a) 3 days pre-op, 7 (sham *n*=12; SNL *n*=16) and 13 days post-op (sham *n*=2; SNL *n*=6). Marked increase in right and left hind paws (b) MTW and (c) TWL difference was observed after SNL, whereas sham surgery did not affect MTW or TWL difference. Statistically significant differences between pre-op and post-op MTW and TWL for each group were observed by Mann–Whitney *U* test (^*∗*^*p* < 0.05, ^*∗∗∗*^*p* < 0.001).

Behavioural assessments (Figure 1(a)) suggest ligation of the left L5 spinal nerve successfully induced neuropathic pain in all animals used in this study. MWL and TWL difference between right and left hind paws were significantly higher at 7 (22.2 ± 2.04 g, 4.57 ± 0.687 s) and 13 days (15.8 ± 2.71 g, 6.11 ± 0.577 s) after SNL, than 3 days before SNL (5.94 ± 2.00 g, −0.389 ± 0.440 s). MWL and TWL difference of sham rats did not differ between the three test points (Figures 1(b) and 1(c)). Thus, mechanical allodynia and thermal hyperalgesia of the left side was evident in SNL rats until their respective endpoints [24].

### 3.2. Macrophage Infiltration and MMP-12 Upregulation Observed in Ligated Spinal Nerves

**Figure 2:**
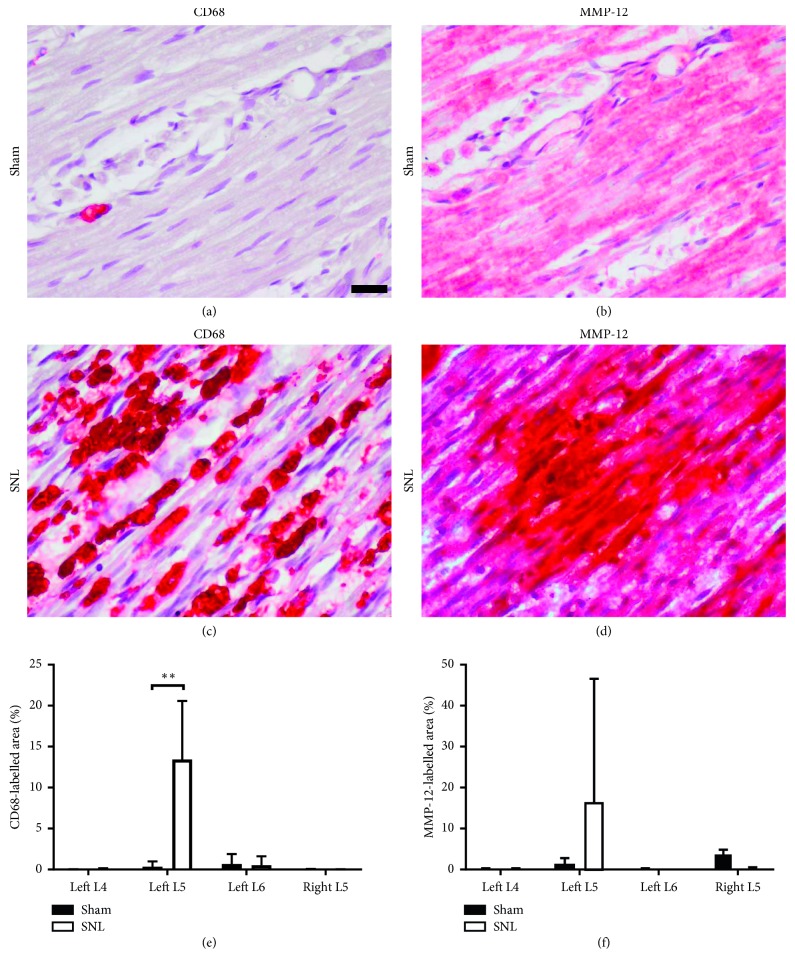
Macrophage infiltration and MMP-12 upregulation occurred in injured nerves. (a–d) Representative images of immunohistochemistry staining on consecutive sections for macrophage marker CD68 and MMP-12, in the left L5 spinal nerves distal to the DRG, 1 week after SNL or sham surgery (CD68 or MMP-12: red, nuclei: purple, scale bar = 20 *μ*m). (e) CD68- and (f) MMP-12-labelled areas were quantified for SNL and sham rats' spinal nerves (*n*=5 per group). Statistically significant difference in the left L5 spinal nerve CD68-labelled area was determined by Mann–Whitney *U* test (^*∗∗*^*p* < 0.01).

**Figure 3:**
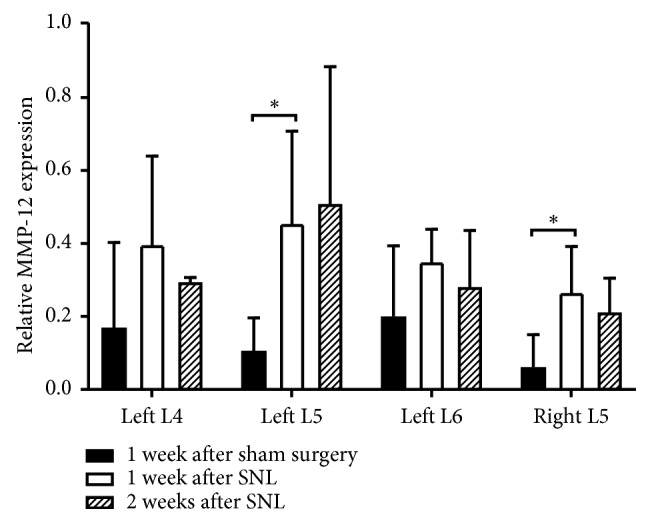
MMP-12 upregulation in ligated left L5 spinal nerve is observed for up to 2 weeks. MMP-12 activity of spinal nerves at 1 (*n*=5) or 2 (*n*=4) weeks after SNL and 1 week after sham surgery (*n*=5) was compared. Statistically significant differences between groups for each spinal nerve were determined by Mann–Whitney *U* test (^*∗*^*p* < 0.05).

One-week postoperative immunostaining for CD68 and MMP-12 showed greater staining in ligated left L5 spinal nerves (CCD68 13.4 ± 7.15%; MMP-12 23.6 ± 18.4%), compared to the left L5 spinal nerves in sham-operated rats (CD68 0.335 ± 0.656%; MMP-12 9.91 ± 9.30%). In SNL rats, CD68 and MMP-12 staining in the left L4, left L6, and right L5 spinal nerves were lower than the left L5 spinal nerve (Figure 2). MMP-12 activity analysis (Figure 3) at 1 week after SNL further confirmed ligation induced significant MMP-12 upregulation in left L5 spinal nerves (0.455 ± 0.253), compared to sham surgery (0.106 ± 0.0900). Moreover, MMP-12 expression remained elevated in left L5 spinal nerves 2 weeks after SNL (0.510 ± 0.374). Hence, the MMP-12-targeted probe was administered 2 weeks after SNL or sham surgery, to ensure animals were fit for intrathecal injection. While sham surgery also induced detectable MMP-12 levels, it is lower than the corresponding spinal nerves in SNL rats. MMP-12 upregulation was observed in spinal nerves contralateral and ipsilateral to the ligated left L5 spinal nerve too. This may be due to local inflammation in response to surgical insult. Nevertheless, MMP-12 levels of nonligated nerves in SNL rats were still lower than ligated nerves. Furthermore, MMP-12 levels in nonligated nerves reduced between 1 and 2 weeks after SNL but increased in ligated nerves, suggesting local inflammation subsidence over time. Taken together, the results suggest that macrophage infiltration occurred primarily at the site of ligation, leading to increased MMP-12 secretion at the pain-generating site [21].

### 3.3. Demyelination and Elastin Fibre Loss Occurred at the Site of Ligation

**Figure 4:**
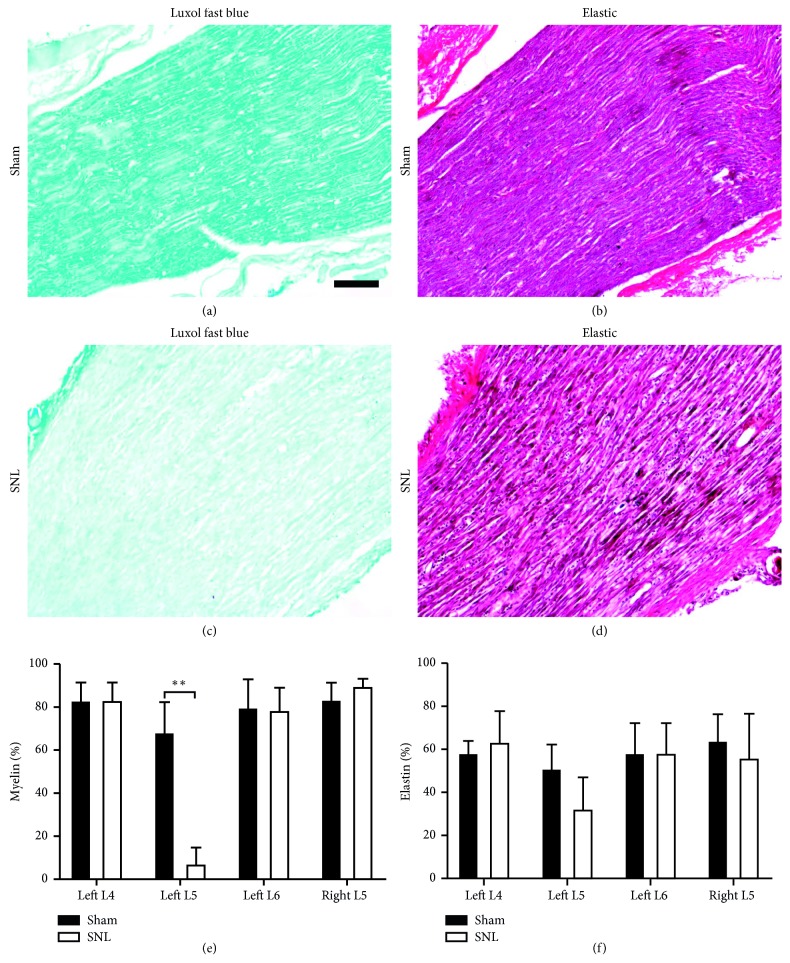
Demyelination and elastin fibre loss occurred at the site of ligation. (a–d) Representative images of Luxol Fast Blue (myelin: blue) and Verhoeff-van Gieson (elastin: black, nuclei: blue, and collagen: red) staining, on consecutive sections of the left L5 spinal nerve distal to the DRG, 1 week after SNL or sham surgery (scale bar = 100 *μ*m). (e) Myelin- and (f) elastin-positive areas of SNL and sham rats' spinal nerves were quantified (*n*=5 per group). Statistically significant difference in the left L5 spinal nerve myelin content was determined by Mann–Whitney *U* test (^*∗∗*^*p* < 0.01).

Significantly lower myelin and elastin fibre content was noted at the site of ligation 1 week after SNL (myelin 19.6 ± 14.5%, elastin 34.1 ± 11.1%), compared to the corresponding tissues of sham rats (myelin 84.5 ± 6.95%, elastin 60.0 ± 4.78%). Demyelination at the site of ligation only is indicative of successful experimental nerve injury (Figure 4) [30]. MMP-12 is known to be the primary enzyme responsible for elastin degradation [31]. Therefore, elastin fibre loss at the site of ligation is in line with MMP-12 upregulation observed in ligated left L5 spinal nerves.

### 3.4. Synthesised Probe Provides Sufficient Contrast *In Vitro*

**Figure 5:**
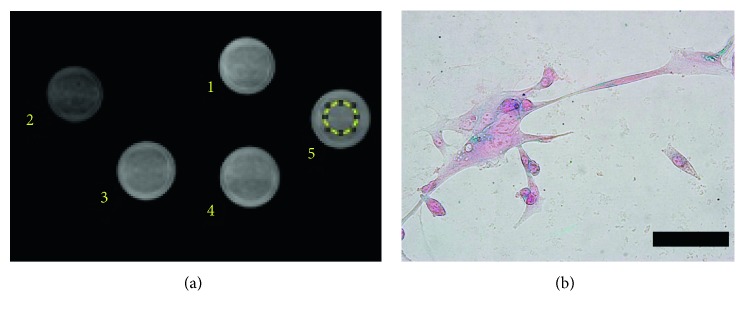
MMP-12-targeted probe is selectively cleaved by MMP-12 *in vitro*. (a) U87 glioma model MRI phantom showed lower intensity in the ROI (indicated by yellow circle) of (2) cells treated with probe only, compared to (3 and 4) cells treated with probe and MMP408, (5) untreated cells, and a (1) blank. (b) Microscopic observation of U87 cells stained with prussian blue, following incubation with the probe, shows iron-positive areas (scale bar = 100 *μ*m).

The optimised IONP had a diameter of 31.9 nm, iron concentration of 1.85 mg, zetapotential of 6.36 ± 8.74 mV, and U87 glioma cell viability of 107%. Furthermore, the relaxivity of our MMP-12-targeted probe (211.62 mmol^−1^s^−1^) is comparable to a commercial contrast agent, resovist (228.83 mmol^−1^s^−1^). Incubating U87 glioma cells with 10 *μ*g/ml MMP-12-targeted IONP resulted in a lower mean grey value (1310 arbitrary units) in the acquired MR image, than cells treated with probe and MMP408 (well 3: 1460 arbitrary units; well 4: 1480 arbitrary units), untreated cells (1460 arbitrary units), and the blank (1500 arbitrary units) (Figure 5). In another set of experiments, 5% MMP408 reduced MMP-12 activity in U87 cells by 40% (0.254 ± 0.0506 ng MMP-12/*μ*g albumin), compared to untreated cells (0.423 ± 0.0315 ng MMP-12/*μ*g Albumin). Hence, the probe is not cytotoxic and is selectively cleaved by MMP-12 *in vitro*, to provide sufficient contrast for MRI [12].

### 3.5. Low MR Signal Observed at the Site of Ligation

**Figure 6:**
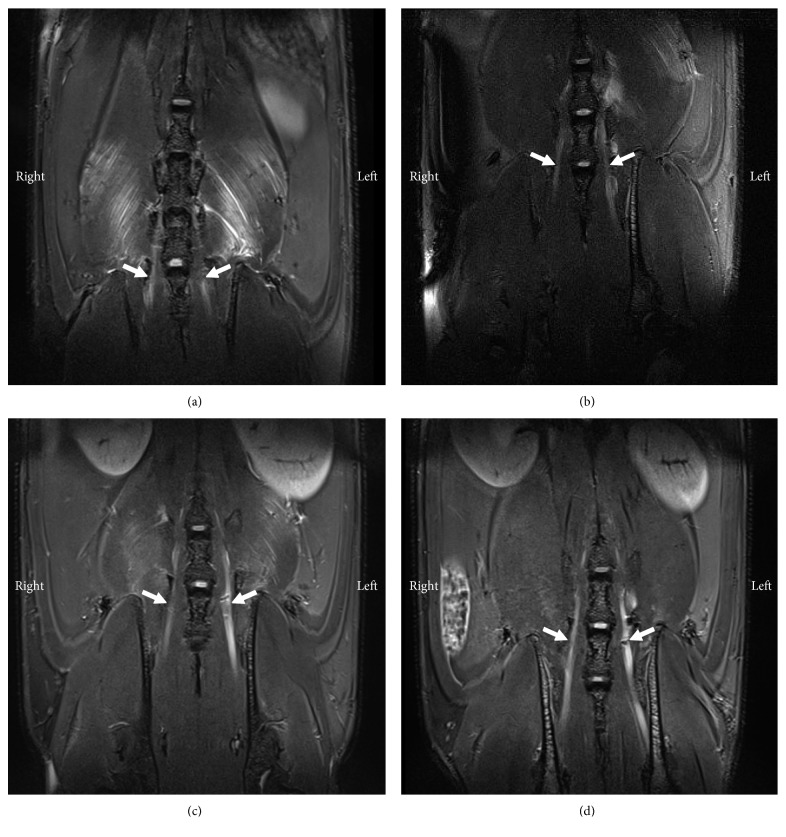
Coronal T2-WI a day after intrathecal injection of MMP-12-targeted probe. White arrows indicate left and right L5 spinal nerves, in sham and SNL rats (*n*=2 per group). Compared to uninjured nerves, injured nerves are enlarged, and iron-induced signal loss is observed at the approximate site of ligation. (a) Sham 1. (b) Sham 2. (c) SNL 1. (d) SNL 2.

**Figure 7:**
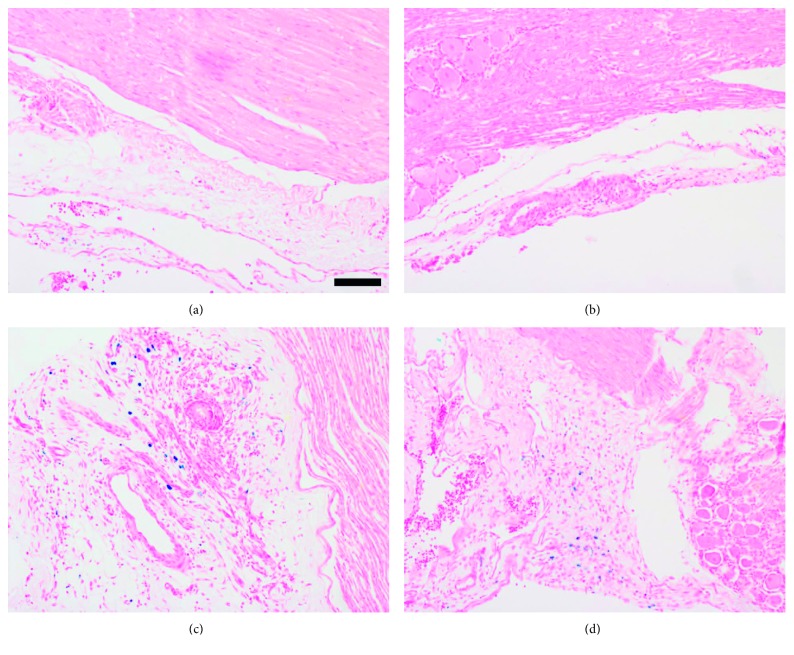
Prussian blue staining verifies MRI observations. Left L5 spinal nerves harvested from SNL rats after MRI show increased iron staining in the perineural tissues, compared to the corresponding tissues in sham rats (*n*=2 per group; iron: blue, nuclei: red, and cytoplasm: pink; scale bar = 100 *μ*m). (a) Sham 1. (b) Sham 2. (c) SNL 1. (d) SNL 2.

In uninjured left and right L5 spinal nerves, the MR signal was slightly hyperintense to the surrounding soft tissue. In comparison, the left L5 spinal nerves of SNL rats were much brighter, and hypointensive areas were observed at the approximate site of ligation (Figure 6). After these rats were terminally perfused, histology of harvested spinal nerves confirmed the presence of iron in injured nerves only (Figure 7). This result suggests that MR signal increase in left L5 spinal nerves of SNL rats is due to ligation and that surgical removal of the left L5 spinus process or the L5 laminar bone did not damage nerves. Moreover, it suggests that only injured nerve tissues would cleave and absorb sufficient MMP-12-targeted IONPs, to cause MR signal decrease.

MMP-12 is implicated in several inflammatory, vascular, and neurological disease pathogenesis [32]. This self-activating enzyme exacerbates inflammation, by activating pro-MMP-2 and pro-MMP-3. These enzymes then activate other MMPs, leading to the degradation of a several ECM proteins [33]. By degrading the ECM, MMP-12 enables macrophage infiltration into injured tissues, for tissue debris phagocytosis. This is an important process in Wallerian degeneration, which occurs before nerve regeneration can begin [34]. In rodent models of sciatic nerve injury, MMP-12 gene and protein levels are elevated [21, 35, 36]. Similarly, we studied MMP-12 protein expression and enzymatic activity in another model of neuropathic pain. We found that elevated MMP-12 in ligated left L5 spinal nerves is associated with macrophage infiltration, ECM degradation, mechanical allodynia, and thermal hyperalgesia. Thus, this model was aptly used for *in vivo* evaluation of the synthesised MMP-12-targeted magnetic IONP.

MMP-targeted probes for *in vivo* imaging of other inflammatory diseases have been developed too. MMPs are well-known biomarkers for cancer, so MMP-targeted magnetic nanoparticles hold promise for enhanced MRI of malignant cell lines and tumour-bearing murine models [37–40]. Apart from injured peripheral nerves, MMP-12 activity upregulation occurs in hind paws of mice with collagen-induced arthritis. This was detected by near-infrared optical imaging, using an MMP-12-selective Forster resonance energy transfer probe [41]. MMP-12 is also a promising target for treatment, as local administration of its selective inhibitor reduces mechanical allodynia and thermal hyperalgesia, a week after partial sciatic nerve ligation [36]. Hence, MMP-targeted molecular probes are valuable tools to improve imaging techniques. They may potentially be used for nerve lesion detection and management, as well as drug development for neuropathic pain.

IONP-based MRI is a feasible technique for clinical use. Some IONPs have been approved as MRI contrast agents, and research on other experimental IONPs is mounting. While MMP-targeted IOPNs have been tested on several disease models, they require further development to increase MR signal detection sensitivity [42]. One means of improving reliability is by developing multitargeted probes. The targeting efficiency of IONPs in injured peripheral nerves may be raised by constructing IONPs that can be cleaved by other upregulated MMPs, namely, MMP-9 and MMP-7 [21]. Other proteins with increased expression in injured nerves include aquaporin-4, interleukin 1 receptor-like 1, and periaxin, which may be detected by antibody-conjugated IOPNs [43].

This is the first study to develop an MMP-targeted nanoprobe for enhanced *in vivo* MRI in a neuropathic pain model, albeit with limitations. To be of clinical utility, contrast agents should ideally be systemically administered. However, developing IONPs suitable for intravenous administration remains a challenge. Biological barriers and interaction with blood proteins limit the amount of systemically circulating IONPs that reach target tissues [19]. To achieve sufficient biodistribution at the lumbar spinal nerves, a large volume of the magnetic particle would have to be injected intravenously. However, this was beyond the scope of the current study. Due to the difficulties of generating the left L5 SNL model [23], sample sizes are small and control experiments using nontargeted IONPs were not done.

## 4. Conclusions

MMP-12 expression is elevated in ligated spinal nerves of a neuropathic pain animal model. Furthermore, MMP-12 is a potential target enzyme for IONP-enhanced MRI of injured spinal nerves.

## Data Availability

The data used to support the findings of this study are available from the corresponding author upon request.
